# COVID-19 Sensing: Negative Sentiment Analysis on Social Media in China via BERT Model

**DOI:** 10.1109/ACCESS.2020.3012595

**Published:** 2020-07-28

**Authors:** Tianyi Wang, Ke Lu, Kam Pui Chow, Qing Zhu

**Affiliations:** 1 Department of Computer ScienceThe University of Hong Kong 25809 Hong Kong; 2 Department of Social Work and Social AdministrationThe University of Hong Kong 25809 Hong Kong; 3 Department of CardiologyQilu Hospital of Shandong University Jinan 250012 China

**Keywords:** COVID-19 sensing, public health, sentiment classification, social media in China

## Abstract

Coronavirus disease 2019 (COVID-19) poses massive challenges for the world. Public sentiment analysis during the outbreak provides insightful information in making appropriate public health responses. On Sina Weibo, a popular Chinese social media, posts with negative sentiment are valuable in analyzing public concerns. 999,978 randomly selected COVID-19 related Weibo posts from 1 January 2020 to 18 February 2020 are analyzed. Specifically, the unsupervised BERT (Bidirectional Encoder Representations from Transformers) model is adopted to classify sentiment categories (positive, neutral, and negative) and TF-IDF (term frequency-inverse document frequency) model is used to summarize the topics of posts. Trend analysis and thematic analysis are conducted to identify characteristics of negative sentiment. In general, the fine-tuned BERT conducts sentiment classification with considerable accuracy. Besides, topics extracted by TF-IDF precisely convey characteristics of posts regarding COVID-19. As a result, we observed that people concern four aspects regarding COVID-19, the virus Origin (Gamey Food, 3.08%; Bat, 2.70%; Conspiracy Theory, 1.43%), Symptom (Fever, 2.13%; Cough, 1.19%), Production Activity (Go to Work, 1.94%; Resume Work, 1.12%; School New Semester Beginning, 1.06%) and Public Health Control (Temperature Taking, 1.39%; Coronavirus Cover-up, 1.26%; City Shutdown, 1.09%). Results from Weibo posts provide constructive instructions on public health responses, that transparent information sharing and scientific guidance might help alleviate public concerns.

## I. Introduction

Coronavirus Disease 2019 (COVID-19), caused by a new coronavirus with higher reproductivity than SARS [1], first emerged in the People’s Republic of China in December 2019 [2]. Early outbreak data grew rapidly at an exponential rate [3], and human-to-human transmission also occurred [4], [5], which brought severe challenges to China and the whole world. Soon vicarious traumatization caused by COVID-19 was found spreading in members of medical teams united in aiding the COVID-19 control and general public [6]. Social media has been found as a key platform for the public on information gathering and social learning to manage uncertainty and risks during a public health crisis. Gui *et al.* [7] investigated public concerns to the Zika virus crisis and reported mechanisms of personal risk assessment and travel-related decision making during the crisis. Meanwhile, social media have been widely used by public health professionals for epidemiological monitoring and understanding public reactions to urgent public health issues. Pei *et al.* [8] developed methods to detect the intensity of social reaction with word-to-vector technique and context analysis. Tibebu *et al.* [9] analyzed real-time information on Twitter about opioid use and perceptions in Canada, which facilitated public health practice and opioid crisis addressing.

To explain and predict public emotional responses, especially the sentiment of distress and grief towards COVID-19, we analyze 999,978 microblogging posts from January 1, 2020 to February 18, 2020 on Sina Weibo^1^ (Weibo for short), one of the most popular social media platforms in China with 550 million monthly active users in Quarter one, 2020. Weibo enjoys its traits of instant messaging, transparent sharing and publicly accessibility. In this work, the Deep Natural Language Processing (NLP) model and topic modelling method are utilized. To be specific, we fine-tune BERT for sentiment classification upon posts with three potential categories of sentiment, positive, neutral and negative, achieving a 75.65% of high accuracy, which surpasses many NLP baseline algorithms. The number of posts on each date is analyzed based on sentiment classification. Thereafter, TF-IDF model is adopted to extract central topics of posts. As the public sentiment on social media reflects people’s psychological well-being and the spread of posts with negative sentiment may lead to social disruption and challenges for infection preventions [10], we analyze 11 dominant and distinctive topics extracted from Weibo posts with negative sentiment and investigate the trends of sentiment development and the underlying major themes to understand public concerns.^1^https://www.weibo.com/

Outbreaks are now taking place in many countries around the world, especially Europe and North America [11]. For instance, as of 25 July 2020, there have been more than 4 million people affected in the U.S. Under this circumstance, main contributions of this study include:

• We fine-tune BERT model for sentiment classification on Chinese Weibo posts about COVID-19 and achieve considerable accuracy that beats all baseline NLP algorithms.
• The study demonstrates how public sentiment on social media evolves as COVID-19 spreads.
• We extract representative topics and discuss the dominant discourse of public distress about COVID-19 caused by related social events. Findings of this study could assist governments worldwide in making efficient and effective public health protection decisions.

## II. Related Work

Coronavirus Disease 2019 (COVID-19) is a newly occurred disease that related research has barely been published by the time of conducting our study. However, there has been some studies on text sentiment classification, which to some extent relate to our work. Ye *et al.* [12] applied Machine Learning SVM [13] model on Chinese product reviews for sentiment (positive or negative) classification and achieved better performance than the classical Semantic Orientation approach. Narayanan *et al.* [14] built a fast sentiment classifier using Naïve Bayes, which achieved 88.80% accuracy on popular IMDB movie reviews dataset. In recent years, researchers have used more deep learning neural network techniques on sentiment classification. Ren *et al.* [15] enhanced word representation with character embeddings and mainly applied CNN for a context-sensitive sentiment classification on Twitter contents. Tang *et al.* [16] proposed sentiment classification upon documents by a combination of LSTM/CNN embedding and gated RNN.

## III. Methodology

**FIGURE 1.**
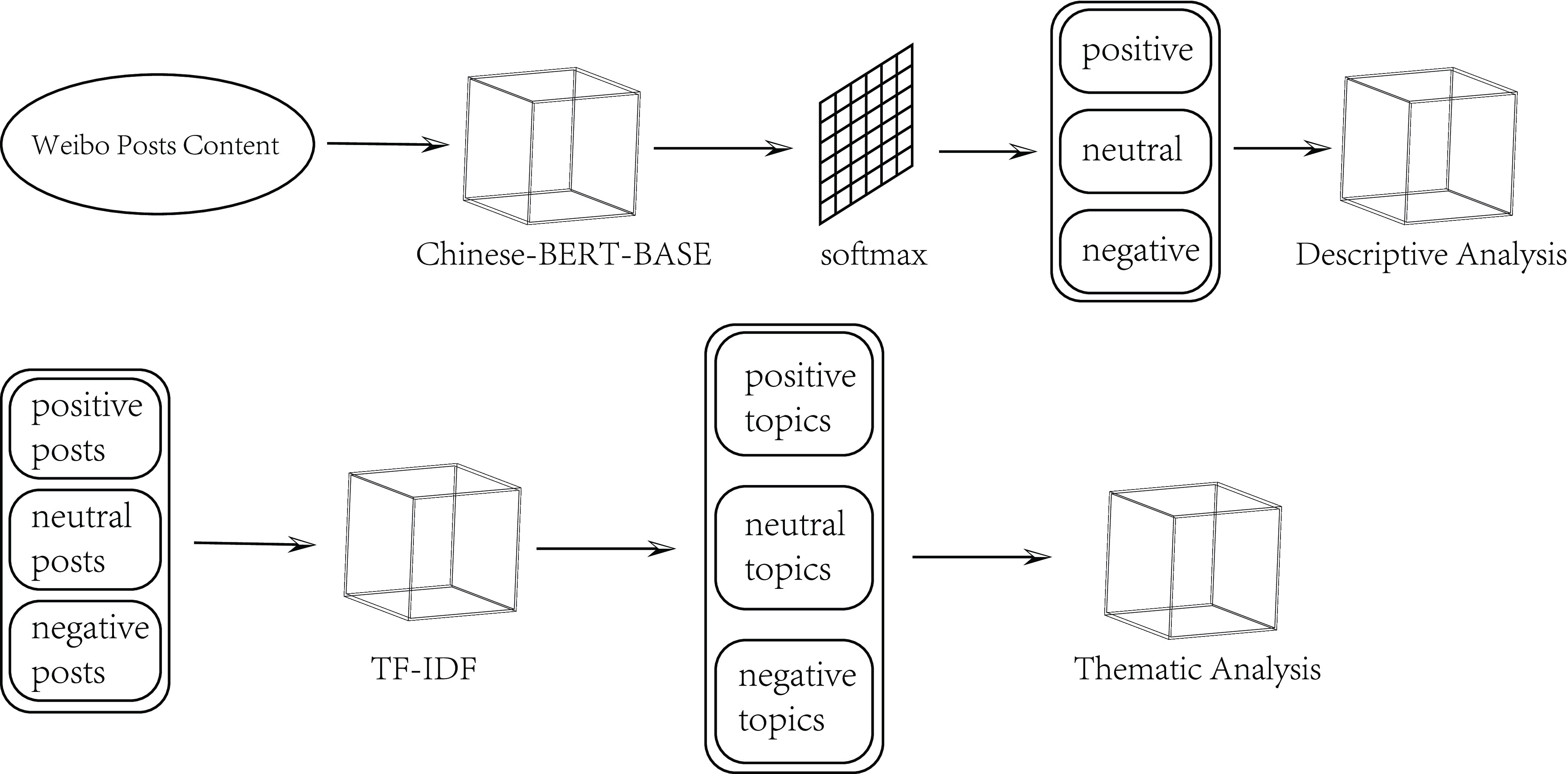
Workflow of sentiment analysis model.

Our sentiment analysis model consists of two parts, as shown in the workflow of Fig. 1. In particular, we first use fine-tuned BERT [17] to classify the sentiment of Weibo posts into positive, neutral and negative categories and analyze the trends of posts. Then we apply TF-IDF [18] algorithm to extract topics of posts with different sentiment. Specifically, 11 topics for negative posts are generalized, and we then analyze the underlying patterns.

### A. Sentiment Classification and Descriptive Analysis

Bidirectional Encoder Representations from Transformers (BERT), a neural network-based technique for natural language processing pre-training, has been largely applied in sentiment analysis [19]. The BERT model can be fine-tuned with proper input and output layers to create state-of-the-art models in a wide range of text analysis tasks [17]. The core of BERT is the adoption of transformer technique [20], which perfectly applies encoder-decoder model [21] and attention mechanism [22]–[24] on NLP tasks. In particular, attention mechanism allows the model to focus on the relevant parts of the input sequence as needed when input sequence is too long for typical NLP models to memorize all input features. In our work, attention mechanism in BERT model helps determine the relevance weight of each word token in input of Weibo posts and generate corresponding hidden states that can best describe the characteristics of different sentiment of Weibo posts during the training process by (1), where $Q$, $K$, and $V$ are three vectors, query, key and value, created based on input embedding, and $d_{k}$ is the dimension of key vectors.

\begin{equation*}\text {Attention}(Q,K,V)=\text {softmax}\left({\frac {QK^{T}}{\sqrt {d_{k}}}}\right)V \tag{1}\end{equation*}

After sentiment classification, the percentage of each sentiment category is calculated. In public health crisis, people’s responses evolve over time [25]. To gain the insights into people’s reactions, Weibo posts with positive, neutral and negative sentiment of each day from 1 January 2020 to 18 February 2020 are compared. Furthermore, in order to identify dates with rapid changes in a number of posts with different sentiment categories, the increasing rate is calculated by (2) and further analyzed according to critical events happened on the corresponding dates.

\begin{align*}\!\!\!\frac {\text {number of posts on a day}\!-\!\text {number of posts on the day before}}{\text {number of posts on the day before}}\!\!\!\!\!\!\!\! \\{}\tag{2}\end{align*}

### B. Topic Extraction and Thematic Analysis

Term frequency-inverse document frequency (TF-IDF) is a numerical statistic reflecting how important a word is to a document in a collection or corpus [26]. The TF-IDF method is able to catch words that occur frequently by calculating term frequency and avoid insignificant words that occur in every document as important by the ability of inverse document frequency. Da Silva and Lopes [27] used TF-IDF to find the most informative Relevant Expression in each document in the corpus of their research. We perform the well-trained automatic sentiment classification model upon the unlabeled Weibo posts, and apply the built-in TF-IDF function in jieba [28], a Chinese word segmentation tool that operates based on its huge pre-trained corpus, to posts in each labeled sentiment class for topic extraction by (3) for word $t$ in a Weibo post $d$ from the entire 999,978 Weibo posts dataset $D$.

\begin{align*} \begin{cases} \text {tf}(t,d)=\text {log}(1+\text {freq}(t,d))\\ \text {idf}(t,D)=\text {log}\left({\dfrac {N}{\text {count}(d\in D:t\in d)}}\right)\\ \text {tfidf}(t,d,D)=\text {tf}(t,d)\times \text {idf}(t,D) \end{cases}\tag{3}\end{align*}

**FIGURE 2.**
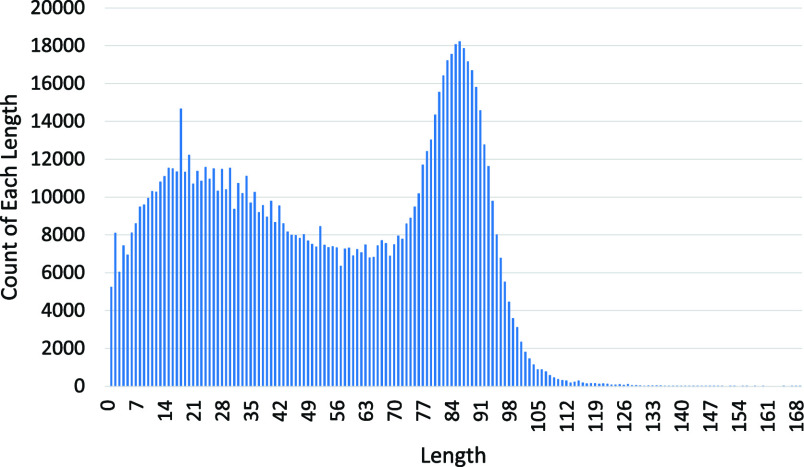
Post lengths of the whole Weibo post dataset. X-axis represents different post lengths, and y-axis represent the numbers of each post length.

In detail, we treat each Weibo post as a document and each segmented Chinese token as a potential topic. Due to the fact that most Weibo posts are not too long (length distribution without an outlier 884 is as shown in Fig. 2), topics with top 5 TF-IDF scores in each post are extracted and analyzed. Topics in each sentiment category represent public focus and concerns regarding COVID-19. Further analysis based on the extracted topics is performed.

Thematic analysis is a common tool to understand the perceptions and reasons for people’s posts with negative sentiment [29]. In our case, topics appearing in more than 1% of total posts in each category of sentiment (positive, neutral and negative) are collected. Five topics are excluded, including Pneumonia (

**Figure:**
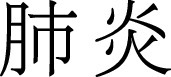

), Outbreak (

**Figure:**
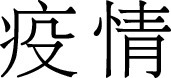

), Virus (

**Figure:**
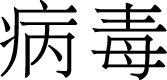

), Coronavirus (

**Figure:**
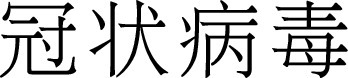

), and COVID-19 (

**Figure:**
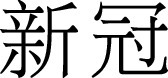

), because they directly represent COVID-19 and couldn’t provide any valuable information in interpreting public sentiment. As a result, there are 38 key topics for positive sentiment, 19 for neutral sentiment, and 19 for negative sentiment, respectively. The 19 key topics for negative sentiment are compared with those of neutral sentiment and positive sentiment. Thereinto, 11 topics are found distinctive for negative sentiment and 8 topics are shared among posts with neutral or positive sentiment, where the latter are Masks (

**Figure:**
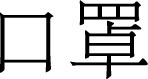

), Wuhan (

**Figure:**
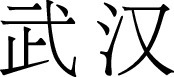

), Definite Diagnosis (

**Figure:**
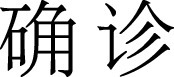

), Doctor (

**Figure:**
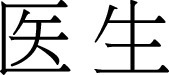

), Case (

**Figure:**
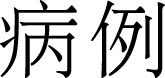

), Infection (

**Figure:**
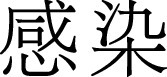

), Quarantine (

**Figure:**
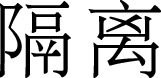

), and Hospital (

**Figure:**
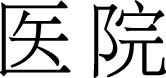

). In addition, by exploring the semantics of topics, we divide the 11 negative-distinctive topics to four themes for further analysis. The four themes are Origin, Symptom, Production Activity, and Public Health Control. Moreover, we analyze the frequency of the 11 topics from 1 January 2020 to 18 February 2020 to visualize their trends.

## IV. Experiments and Results Analysis

### A. Dataset

Based on a list of 230 COVID-19 related key phrases, 2.4 million Weibo posts from 1 January 2020 to 19 February 2020 (posts on 19 February is incomplete thus excluded in our final sample) are crawled by CCIR 2020 organizer (26th China Conference on Information Retrieval) [30]. The crawler mainly uses SciPy and Beautiful Soup techniques, and deletion of duplicates and reposts are processed to construct the Weibo posts dataset. The dataset includes posts by around 640 thousand users with user location information excluded.

Manual sentiment labelling (positive, neutral and negative) is accomplished by CCIR 2020 organizer. In particular, open source Chinese sentiment analysis tools are adopted for preprocessing, and 12 volunteers were invited to finish the manual labeling on 120 thousand randomly selected Weibo posts from the dataset based on the preprocessing results and human decisions upon sentiment of Weibo contents. In detail, label of each Weibo post is decided by majority voting method of 3 volunteers who didn’t know each other.

As a result, one million randomly selected posts from the whole dataset are shared to the public and 10% of them are manually labeled with three sentiment categories (positive, neutral and negative) by CCIR 2020 organizer.

### B. Sentiment Classification

Manually labeled Weibo posts are randomly split into training and testing sets with a ratio of 5 to 5. In our experiment, we fine-tuned the Chinese BERT-BASE model with 12 layers and hidden dimension 768. According to the suggestion on hyperparameters selection by the original BERT paper [17], we set up parameters of 4 epochs, learning rate of 2e-5 and batch size of 32, and applied softmax neural network layer to train a three-category (positive, neutral and negative) sentiment classifier using the training dataset.

After training, the sentiment classification model achieves a 75.65% accuracy upon testing set. F1-scores on testing set along with precision and recall metrics for each sentiment category are summarized in Table 1. An overall weighted F1-score 0.7458 for classification model is obtained on data in Table 1 by (4), where $w$ is the weight of each sentiment category. The sentiment classification is then performed upon the rest unlabeled Weibo posts.

\begin{align*} \begin{cases} F_{1}=\dfrac {2}{\text {recall}^{-1}+\text {precision}^{-1}}\\ \text {Weighted } F_{1}= w_{\text {positive}} \times F_{1,\text {positive}}\\ \qquad \qquad \qquad +w_{\text {neutral}}\times F_{1,\text {neutral}}\\ \qquad \qquad \qquad +w_{\text {negative}}\times F_{1,\text {negative}} \end{cases}\tag{4}\end{align*}

**TABLE 1:** Performance Measures of Sentiment Classification Model

	Precision(%)	Recall(%)	F1-score(%)
Positive	0.6990	0.7477	0.7225
Neutral	0.7975	0.7797	0.7885
Negative	0.6434	0.6246	0.6339

We performed comparative tests on the same dataset using different baseline sentiment classification algorithms with the same data split ratios and random state values. Results are presented in Table 2.

**TABLE 2:** Comparative Test on NLP Baseline Algorithms

System	Accuracy(%)
Fine-tuned $\text {BERT}_{\text {BASE}}$	75.65
SVM	70.66
Naïve Bayes	66.97
Logistic Regression	70.02
CNN	71.19
LSTM	57.73

**FIGURE 3.**
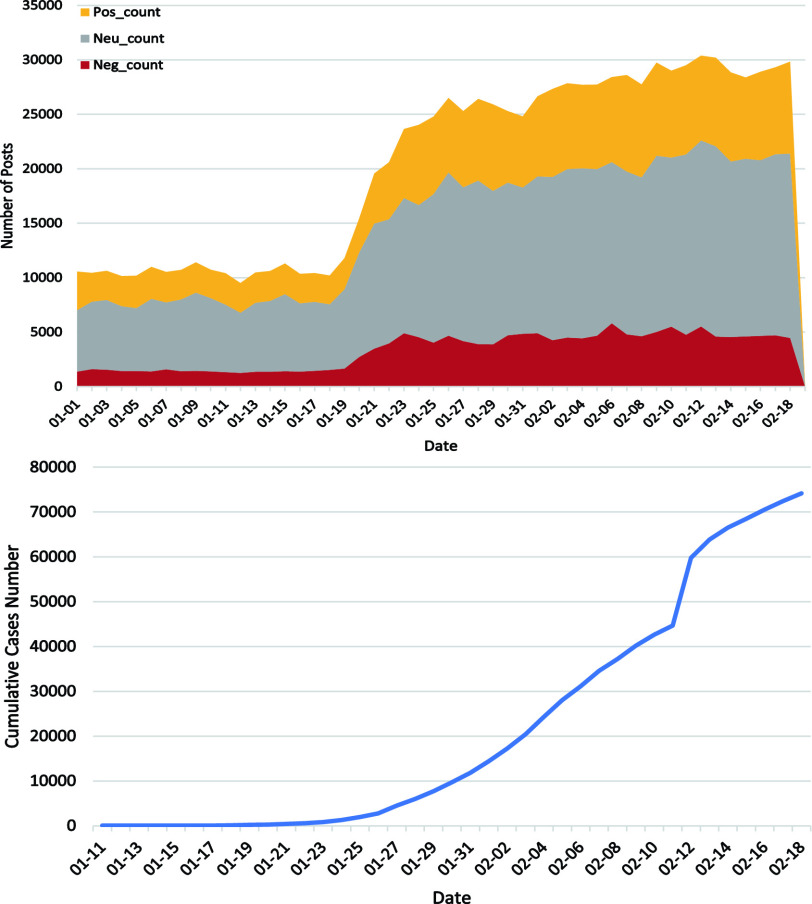
Posts with three different sentiment categories with respect to the dates (upper graph) and number of accumulative confirmed cases with respect to the dates (lower graph).

For 999,978 posts, most of them (56.2%) are neutral, while there are more positive sentiment (27.4%) than negative sentiment (16.4%). Examples of posts with different sentiment categories are given in Table 3. Specifically, posts of expressing gratitude would be labelled as positive while those about the fear for COVID-19 or life arrangements affected by COVID-19 would be categorized as negative. Neutral posts include the mere circulation of public information or plain descriptions of some life changes. From the upper part of Fig. 3, it is clear that COVID-19 related posts are increasing across the time and posts with different sentiment categories increase accordingly. Before 19 January, the number of posts about COVID-19 are quite stable. As to 20 January, there is an increase of 25.81% and 33.03% in negative posts and total posts, respectively, and a decrease of 16.30% in positive posts as compared to that of 19 January. Regarding the accumulative confirmed cases of COVID-19 in lower part of Fig. 3, it is clear that the number of posts in each day starts to grow from 20 January, in line with the increase in accumulative confirmed cases. The number remains relatively stable from early February during the early outbreak. COVID-19 is first noticed in China in December 2019 [1], but the number of Weibo posts remains stable and relatively low during the early period. On 20 January 2020, there is a surge in total Weibo posts and posts with negative sentiment as well, and the number of posts is kept at that level ever since. One important event might be related to this. On the night of 20 January Dr. Zhong Nanshan confirmed the human-to-human transmission of COVID-19 on China Central Television [31]. This surge of Weibo posts indicates the tremendous influence of information revealed by government-related media and it is therefore important for government to discreetly handle the public depression about COVID-19.

**TABLE 3:** Examples of Posts With Different Sentiment Categories

Sentiment	Post
Positive	#, #Salute to the warriors wearing white clothes (the doctor)# Thanks for your hard work. Stay strong China, stay strong Wuhan, I wish all the best for my hometown.
Neutral	COVID-19 WHO officially name the new coronavirus as COVID-19. $\ldots \ldots$? The view of my bedroom window is the often-spacious garden walkway of the neighborhood. There used to be people running or walking. However, after a confirmed case being announced in our community, it’s hard to see even a single person.
Negative	38.5? Finally getting out of hospital and now I’m having a fever with 38.5°C. What’s wrong with me, what’s wrong with me? So boring. Work Resumption has been postponed again. I’m so worried the company will be gone one day.

### C. Negative Sentiment Analysis

**FIGURE 4.**
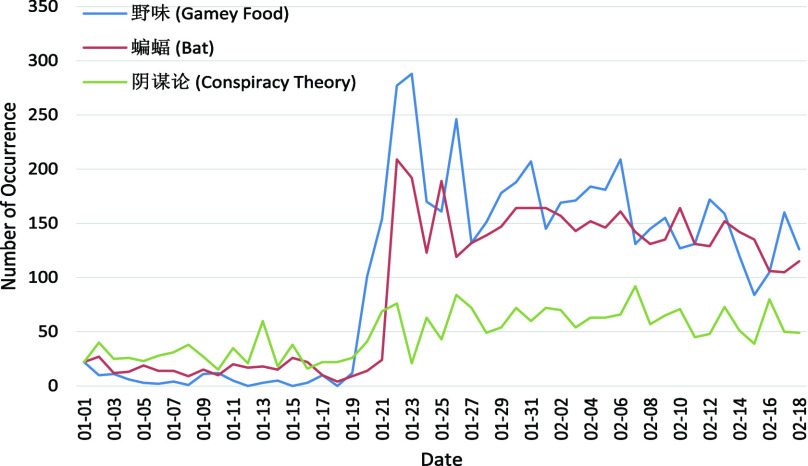
Number of posts related to the Origin with respect to the dates.

**FIGURE 5.**
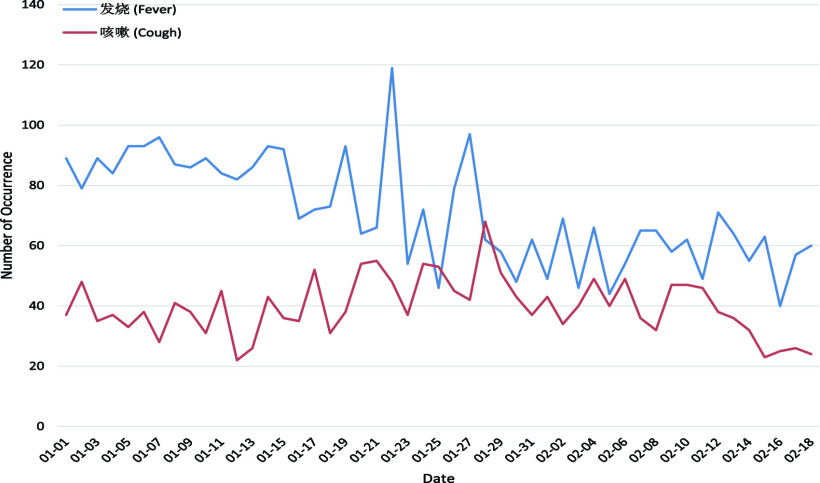
Number of posts related to the Symptom with respect to the dates.

**FIGURE 6.**
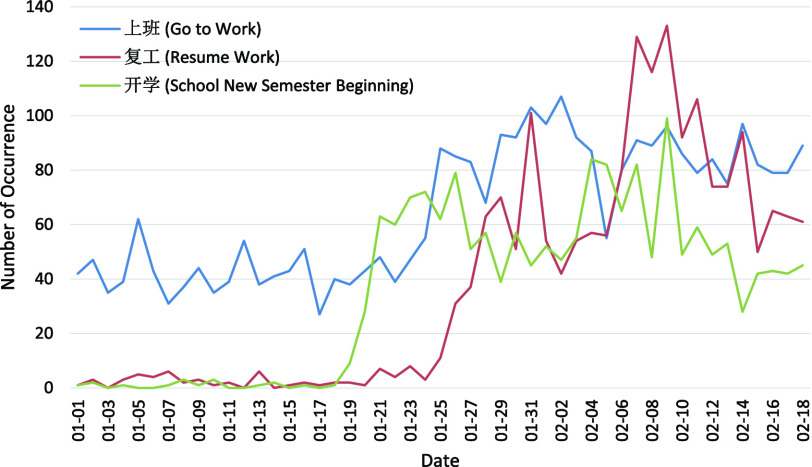
Number of posts related to the Production Activity with respect to the dates.

**FIGURE 7.**
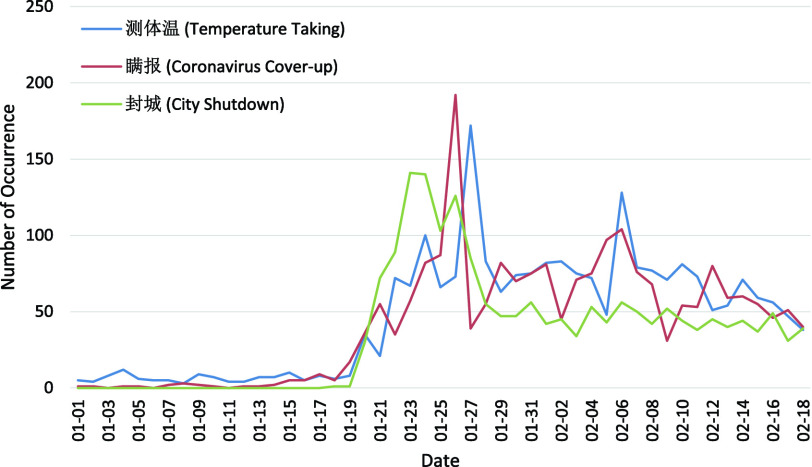
Number of posts related to the Public Health Control with respect to the dates.

As listed in Table 4, the 11 key topics of Weibo posts with negative sentiment fall into four themes: Origin, Symptom, Production Activity, and Public Health Control. Typically, the number of posts in each theme for each day from 1 January 2020 to 18 February 2020 are plotted in Fig. 4–7 for further analysis.

**TABLE 4:** 11 Key Topics in Weibo Posts With Negative Sentiment Along With the Corresponding Themes and Frequency of Occurrences in Weibo Posts

Key Topics	Themes	Percentage(%)
(Gamey Food)	Origin	3.08
(Bat)	Origin	2.70
(Conspiracy Theory)	Origin	1.43
(Fever)	Symptom	2.13
(Cough)	Symptom	1.19
(Go to Work)	Production Activity	1.94
(Resume Work)	Production Activity	1.12
(School New Semester Beginning)	Production Activity	1.06
(Temperature Taking)	Public Health Control	1.39
(Coronavirus Cover-up)	Public Health Control	1.26
(City Shutdown)	Public Health Control	1.09

The definitions of four themes in this work are as follows:
Origin: Discussions about where COVID-19 comes from. Symptom: Change of body or mind indicating the possible infection of COVID-19. Production Activity: Work life arrangements influenced by the outbreak of COVID-19. Public Health Control: Measures to stop the spread of COVID-19.

There are three topics about the Origin of COVID-19 as shown in Fig. 4. In particular, “Gamey Food” (3.08%) and “Bat” (2.70%) are two primary assumptions for the origins of COVID-19 [5], [32]. They remain low frequency in the early January. However, there is a sharp increase in posts of “Gamey Food” on 20 and “Bat” on 22, January 2020, both then keeping a high frequency thereafter and reaching the highest number of posts with negative sentiment per day among 11 topics. And the “Conspiracy Theory” (1.43%) suggesting that COVID-19 does not have a natural origin is condemned by scientists [33] but widespread on social media. However, it remains in a relatively low frequency in comparison with “Gamey Food” and “Bat”. The discussions of the origins of virus are deeply correlated to negative sentiment and trigger the largest amount of posts with negative sentiment as demonstrated in Fig. 4. However, as discussed, rumors and unconfirmed information may overwhelm the discussion [33]. Consequently, it is important for the government to release the transparent progress about the investigation of origins.

“Fever” (2.13%) and “Cough” (1.19%) are identified as the representative symptoms of COVID-19 [34]. For posts about Symptom as shown in Fig. 5, posts with “Fever” as topic outnumbered that with “Cough”, and the gap is quite large in early January but declines in February as COVID-19 spread. Symptoms about COVID-19 might lead to negative sentiment but could also be beneficial for self-detection of infection. Therefore, typical symptoms of disease should be revealed to the public clearly and timely, which would benefit early detection of the disease.

Production Activity as pictured in Fig. 6 summarizes topics about work-life arrangements under the threat of COVID-19. “Go to Work” (1.94%) and “Resume Work” (1.12%) portray the public concern over work, and “School New Semester Beginning” (1.06%) indicates people’s worries about students going back to school. The concern on “Go to Work” starts from early January and remains relatively high while the one on “School New Semester Beginning” starts to grow from 20 January 2020 and the worries for “Resume work” starts to grow from 26 January 2020. The arrangements for production activity might be an important driving force for the general depression. The peak of concern for “School New Semester Beginning” in Fig. 6 comes earlier than that for “Resume Work”, thus earlier arrangements are necessary in easing people’s tension.

Three topics about Public Health Control (Fig. 7) depict different aspects. “Temperature Taking” (1.39%) is an important method for COVID-19 diagnosis. “Coronavirus Cover-up” (1.26%) is the misbehavior or fault in public health control which might lead to risk of more infections [35]. And “City Shutdown” (1.09%) describes the strict public health control taken by the Chinese government [36]. There are almost no negative posts about Public Health Control in the early January. The peak discussions are “City Shutdown” the first on January 23, “Coronavirus Cover-up” the second on January 26, and “Temperature taking” the third on January 27, and the three topics reach the same level of attention around 18 February 2020. This trend demonstrates the focuses on different control measures as situation develops. Towards this end, the public health control and opacity of information may also lead to people’s depression. Therefore, the corresponding public health control measures at a certain time period should be elaborated for public acceptance.

## V. Conclusion

The spread of COVID-19 has turned to a worldwide pandemic thus far [37]. Public health concerns not only relate to the infection prevention but also the psychological status of people experiencing the disaster [38]. Therefore, analyzing posts with negative sentiment from social media could contribute to understanding the experiences of Chinese general public during the outbreak of COVID-19 and offers examples for other countries. Our analyses provide insights on the evolution of social sentiment over time and the topic themes connected to negative sentiment of Weibo posts. Fig. 3 illustrates the clear outbreak dates for public attention about COVID-19. Moreover, concerns about Origin, Symptom, Production Activity, and Public Health Control are deeply intertwined with the public sentiment.

This study collects data on social media from early stage of COVID-19 transmission in China. Based on the data analysis and discussion, several advantages emerge. First, state-of-the-art fine-tuned BERT classification model and TF-IDF topic extraction model deliver results with considerable accuracy. Second, it can further be implemented as an online platform for real-time monitoring on public sentiment during other crises in the future. Third, this study reveals important topic themes which are deeply connected to sentiment of depression. As the infection of COVID-19 keeps spreading worldwide now, insights from this study may contribute to public administration and prevention of social disruptions.

Despite of informative results found in this study, further improvements are expected on the classification model to achieve a higher accuracy. Furthermore, only information on Sina Weibo is used in this study, which may lead to bias by neglecting posts on other social media platforms. Finally, in order to focus on the centrality of topics, only topics appearing in more than 1% of total posts are selected in each category of sentiment. This may lead to the overlook of important topics with less percentage. Future studies by incorporating information in empirical data from different social media platforms and different countries may contribute to a more solid conclusion.

New outbreaks are taking places in many other countries all around the world. The sentiment classification model and findings of this study would provide constructive instructions for governments worldwide on making efficient and effective public health protection decisions.
